# Service user involvement in mental health service commissioning, development and delivery: A systematic review of service level outcomes

**DOI:** 10.1111/hex.13788

**Published:** 2023-06-08

**Authors:** Naseeb Ezaydi, Elena Sheldon, Alex Kenny, Elizabeth Taylor Buck, Scott Weich

**Affiliations:** ^1^ School of Health and Related Research The University of Sheffield Sheffield UK; ^2^ The McPin Foundation London UK

**Keywords:** coproduction, mental health, patient involvement, service improvement

## Abstract

**Introduction:**

Service user involvement is increasingly considered essential in mental health service development and delivery. However, the impact of this involvement on services is not well documented. We aimed to understand how user involvement shapes service commissioning, development and delivery, and if/how this leads to improved service‐level outcomes.

**Methods:**

A systematic review of electronic databases (MEDLINE, PsycINFO, CINAHL and EMBASE databases) was undertaken in June and November 2022 for studies that incorporated patient involvement in service development, and reported service‐level outcomes. Included studies were synthesised into a logic model based on inputs (method of involvement), activities (changes to service) and outputs (indicators of improvement). PRISMA (Preferred Reporting Items for Systematic Reviews and Meta‐Analysis) guidelines were followed when conducting this review.

**Results:**

From 10,901 records identified, nine studies were included, of which six were judged to have used co‐production or co‐design approaches. Included studies described service user involvement ranging from consultation to co‐production. We identified a range of outputs associated with service user involvement in service planning and delivery, and reported these in the form of a logic model. These service‐level outputs included improved treatment accessibility, increased referrals and greater service user satisfaction. Longer‐term outcomes were rarely reported and hence it was difficult to establish whether outputs are sustained.

**Conclusion:**

More extensive forms of involvement, namely, co‐design and co‐production, were associated with more positive and substantial outputs in regard to service effectiveness than more limited involvement methods. However, lived experience contributions highlighted service perception outputs may be valued more highly by service users than professionals and therefore should be considered equally important when evaluating service user involvement. Although evidence of longer term outcomes was scarce, meaningful involvement of service users in service planning and delivery appeared to improve the quality of mental health services.

**Patient or Public Contribution:**

Members of a lived experience advisory panel contributed to the review findings, which were co‐authored by a peer researcher. Review findings were also presented to stakeholders including service users and mental health professionals.

## 1 INTRODUCTION

**Figure 1:**
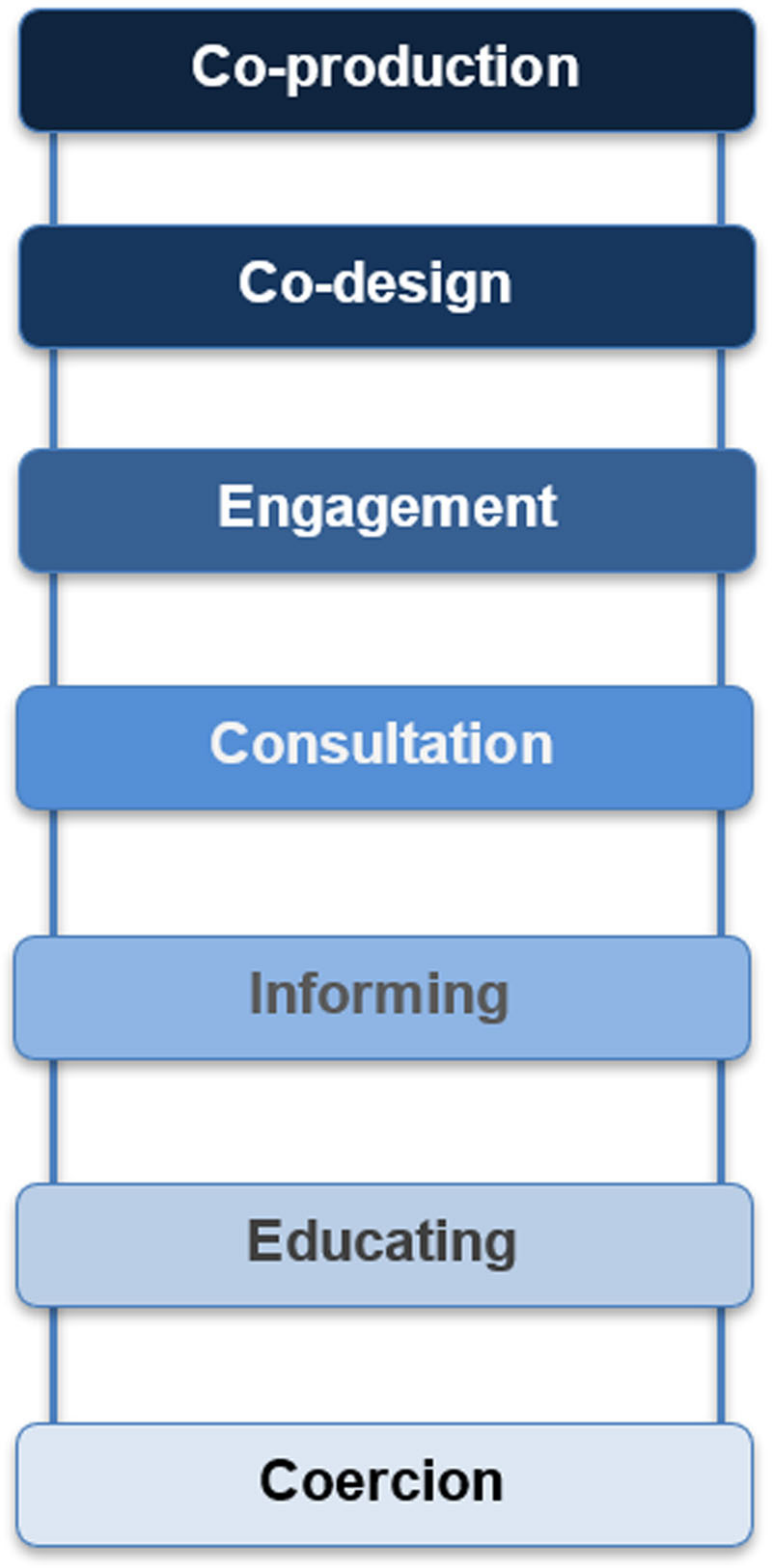
Ladder of co‐production. Figure adapted from NCAG.^2^

Service user involvement is increasingly seen as essential to the effective functioning of healthcare systems.^1^ The National Co‐production Advisory Group (NCAG) published a ladder of co‐production that details a hierarchy of patient involvement and associated methods (Figure 1).^2^ The bottom rungs represent passive participation, where power holders aim to educate or at worst subject service users to coercion.^3^ In the middle of the ladder, service users are involved as advisors or participants and their contributions take the form of feedback; for instance, through surveys or one‐off consultations.^4^ Upper rungs represent involvement approaches that increase service users' decision‐making power such as co‐production.^3^ Co‐production gives full weight to service users' contributions and values their expert experience alongside healthcare professionals' knowledge.^4^ Co‐production at the highest level requires an equal partnership between service users and providers in service development and delivery.^3^

Service user involvement has been described at three levels: microlevel (service users making decisions about their own care), mesolevel (service user representation at the healthcare service level) and macrolevel (service user involvement in healthcare policy and legislation).^1^ This review focuses on the mesolevel within mental healthcare services, in both acute and community settings.

The National Collaborating Centre for Mental Health identified six core principles of co‐production, using the acronym CARING: *C*elebrate involvement, *A*daptable, *R*esources, *I*nfluence of power, *N*eeds‐led and *G*rowth.^5^ They also described three levels of involvement: ‘doing with’, ‘doing for’ and ‘doing to’.^5^ The report encourages a shift from ‘doing for’ towards ‘doing with’ in mental health commissioning by addressing barriers to co‐production such as (limited) staff engagement, lack of resources and confusion about expected contributions.^5^ The report also identified an evidence gap in the outcomes and benefits of co‐production within mental health services.^5^

Existing literature has reported the beneficial effects for service users involved in the co‐production process at the mesolevel, including increased empowerment and agency, reduced stigma and positive impacts on self‐esteem and identity.^6^ However, these benefits may not translate to users of co‐produced services or to the services themselves in terms of outcomes such as cost‐effectiveness and reductions in health inequalities.^6^, ^7^ As well as complexity arising from heterogeneity in co‐production approaches, previous studies are difficult to synthesise because they are mostly based on case studies and concerned with the experiences of co‐production participants rather than the effects of co‐produced changes on service user or service‐level outcomes.^6^, ^8^, ^9^ There is currently no consensus about how the latter should be assessed.^9^, ^10^

The aims of this review were to establish whether and how service user involvement in service commissioning, development and delivery leads to improved outcomes, including at the service level. This was done by undertaking a systematic review of studies in which mental health services were commissioned, developed or delivered in ways that involved service users, and which evaluated service user and/or service‐level outcomes. Findings were synthesised in the form of a logic model showing inputs (involvement methods), activities (changes to service), outputs (early signs of change) and outcomes (intermediate or longer term impacts). We sought to elucidate associations between co‐production methods and outputs and/or outcomes. We hypothesised that there would be a positive association between the extent of service user involvement and improved outcomes for service users and services.

### 1.1 Review question

Does service user involvement in service commissioning, development and delivery lead to improved service level outcomes?

### 1.2 Objectives

1. Conduct a systematic search of databases for studies in which mental health services were commissioned, developed or delivered in ways that involved service users, and which evaluated service user and/or service‐level outcomes.
2. Map identified methods of involvement onto a logic model of inputs (involvement methods), activity (changes to service), outputs (early signs of change) and outcomes (long‐term impacts).

## 2 METHODS

A systematic review was conducted to answer the research question. The review was conducted and reported according to PRISMA (Preferred Reporting Items for Systematic Reviews and Meta‐Analysis) standards (Supporting Information: FS1).^11^ The review protocol was not registered.

### 2.1 Search strategy

The following databases were systematically searched in June 2022: MEDLINE, PsycINFO and CINAHL. An additional database (EMBASE) was later searched in November 2022. Database selection was based on previous reviews investigating co‐production.^9^, ^10^, ^12^ Databases were searched for relevant papers using key search terms identified through relevant reviews and primary studies during an initial scoping search. There were no limitations concerning dates. The full search strategy can be found in the supporting information (Supporting Information: FS2). Database searches were conducted by a single reviewer. Further papers were identified through the reference lists of eligible studies and by citation tracking of eligible studies using Google Scholar.

### 2.2 Eligibility

Rayyan (an online study selection tool)^13^ was used to input database search results. Database search results were initially filtered by screening titles and abstracts for relevance. A second reviewer independently screened 10% of identified records. Full texts of potentially relevant papers were obtained and appraised to identify studies which met the full inclusion and exclusion criteria (Supporting Information: FS3).

Initial scoping identified a paucity of papers and thus a broad eligibility framework was used to include both qualitative and quantitative outcomes. Additionally, there were no restrictions on study design or date of publication (the end date of the search was 13 June 2022 [MEDLINE, PsycINFO and CINAHL] and 21 November 2022 [EMBASE]).

Service user involvement was defined as any form of activity that involved participation in the commissioning, design, monitoring, development and delivery of mental health services. This was restricted to mental health services, in either acute or community settings. Involvement in research or participation in individual treatment choices within patient‐provider consultations was excluded. Only papers published in English were included.

Outcomes were limited to service user and service‐level outcomes in the mental health services described in included studies. These outcomes included changes in service organisation and structure, attendance, accessibility, service user‐reported satisfaction and any health‐related indicators. Outcomes regarding service user or staff perspectives on the involvement process itself were not included.

### 2.3 Quality assessment

Quality assessment was carried out using the Mixed Methods Appraisal Tool version 2018, to assess the methodological quality of eligible studies (Supporting Information: FS4).^14^ This tool is designed for systematic reviews which incorporate quantitative, qualitative and mixed methods studies and was therefore appropriate for assessing papers included in this research. For each study, a single reviewer assessed each criterion (‘Yes’, ‘No’ or ‘Can't tell’) within the appropriate study design category.

### 2.4 Data extraction

Data extraction was completed by two reviewers using a data extraction form that was piloted on a subsample of eligible papers to ensure it met the purposes of this review (Supporting Information: FS5). Information extracted included study aims, nature of service, population served, involvement methods, changes to service (outputs) and outcomes produced as a result of involvement.

### 2.5 Synthesis

Quantitative and qualitative data were synthesised to determine how different outcomes are enabled by different involvement strategies in different contexts. This allowed an exploration of the ‘dose‐response’ effect by investigating whether more meaningful co‐production led to better service level outcomes. This was structured by mapping identified methods of involvement onto a logic model, consisting of inputs (involvement methods), activities (changes in services), outputs (early signs of change to service‐level outcomes) and outcomes (long‐term impacts). Once this information was mapped, links between activities and outputs were identified.

Each study was characterised according to the involvement approach using NCAG's ladder of co‐production (Figure 1). This was corroborated and refined in collaboration with a lived experience advisory panel.^2^ This allowed discussion about whether lesser forms of involvement are necessarily limited in the outcomes they can achieve. Narrative synthesis was used to discuss similarities and differences in studies regarding their inputs and outputs.

### 2.6 Patient or public contribution

This review was co‐authored by a peer researcher and received input from members of a lived experience research advisory panel. The panel assisted with the categorisation of studies against the ladder of co‐production.^2^ The different rungs of the ladder and what they represented were first explained to the panel. Following this, the involvement methods reported in each paper were discussed in detail. Participants discussed and ultimately agreed on which rung of the co‐production ladder each best fit. These judgements were compared with the prior ratings arrived at by researchers. The panel's consensus on 10 out of the 11 methods presented matched the researcher's judgement. The paper in which there was disagreement was then discussed in further detail, and agreement was reached with the researcher's initial judgement. The panel's interpretation of the outputs also informed this review's discussion. Review findings were also presented to a variety of stakeholders including service users and mental health professionals.

## 3 RESULTS

### 3.1 Search results

**Figure 2:**
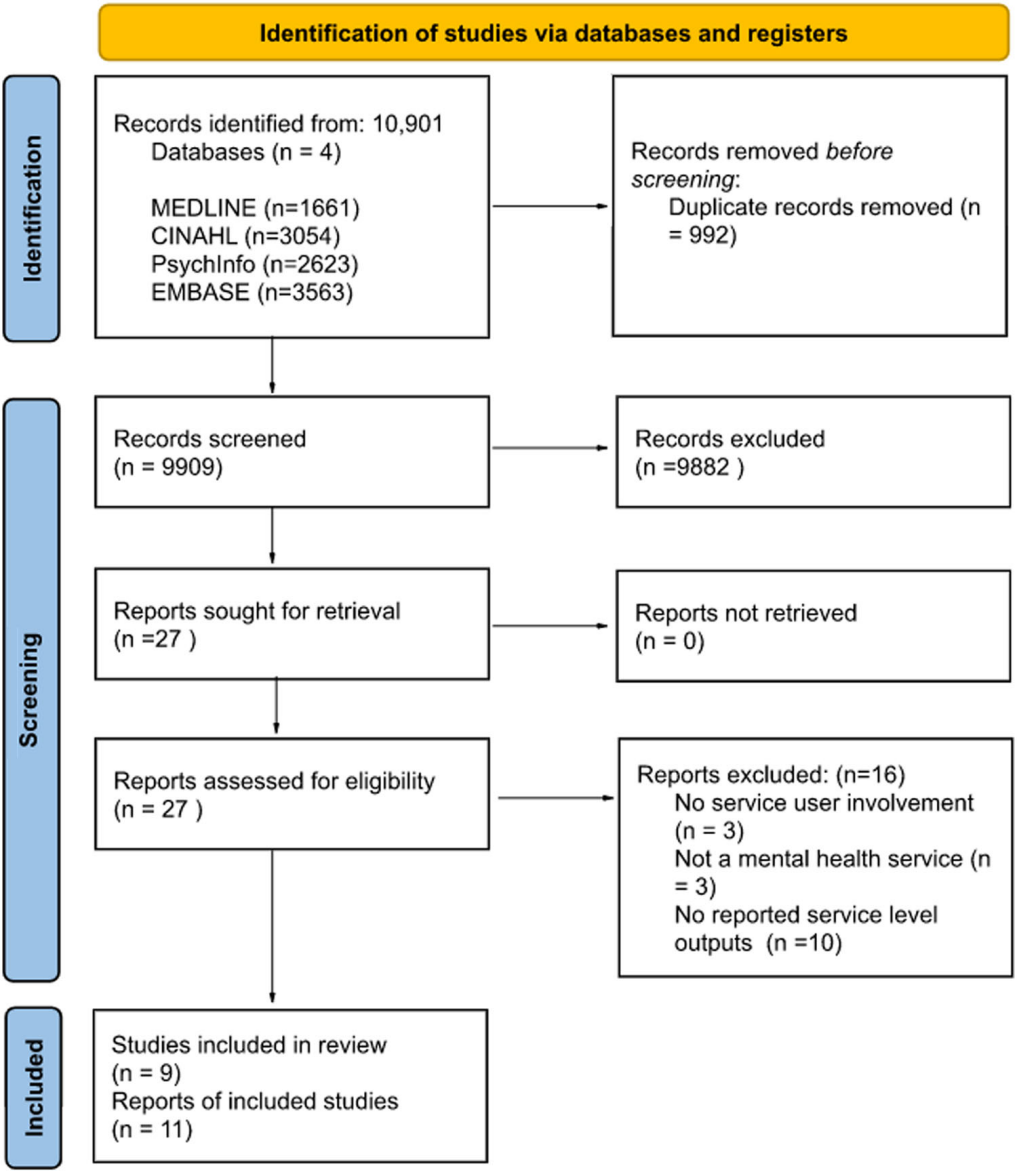
PRISMA (Preferred Reporting Items for Systematic Reviews and Meta‐Analysis) flow diagram to show the process of study selection.

Implementation of the focused search strategy yielded 10,901 records across four databases (MEDLINE, PsychInfo, CINAHL and EMBASE). Of these, 992 duplicates were detected and removed, and 9882 records were excluded at the title stage. A total of 122 abstracts were screened, and from these 27 full texts were assessed for eligibility against the inclusion/exclusion criteria (Supporting Information: FS3). Sixteen studies were rejected after full‐text appraisal, the main reason being the absence of reported service‐level outputs (Supporting Information: FS6). A total of nine studies were included in the final review. One of these studies encompassed three papers,^15^, ^16^, ^17^ from which relevant data on involvement and outputs were extracted. This group of studies will be referred to as the Jigsaw study. Citation tracking and reference list searches yielded no further eligible studies. The following PRISMA flow diagram outlines the stages at which records were excluded (Figure 2).

### 3.2 Study characteristics

A summary of study characteristics is detailed in Table 1. There is significant heterogeneity in the included studies in regard to participants, services, methods and outputs. A range of services was included, with the majority (six out of nine) being community mental health services and the remaining being hospital mental health services (three out of nine). Two of the services specifically catered for young people,^15^, ^16^, ^17^, ^22^ one service targeted those aged over 65 years,^20^ one targeted those with learning disabilities^25^ and another was aimed at black and minority ethnic communities.^23^ Two studies described service user involvement in implementing new services,^15^, ^16^, ^17^, ^22^ whereas the remainder focused on improving pre‐existing services. Three studies adopted a mixed methods design in which quantitative and qualitative data were collected.^18^, ^20^, ^21^ The most common methods used were questionnaires,^15^, ^16^, ^17^, ^18^, ^19^, ^20^, ^21^, ^22^ focus groups^18^, ^23^ and interviews.^21^, ^23^, ^25^ One study was a randomised controlled trial,^19^ three were quantitative studies,^16^, ^22^, ^24^ two were qualitative in design^23^, ^25^ and three employed mixed methods.^18^, ^20^, ^21^

**Table 1:** Characteristics of included studies.

References	Country	Study design	Service type	Population served	Study objective	Method of involvement	Outputs reported
Pocobello et al.^18^	Italy	Mixed methods study Qualitative focus groups and quantitative cross‐sectional study.	Coproduced community mental health day centre which organises activities, support groups and so on.	Members with lived experiences of mental health issues.	Investigate the differences between a co‐produced experimental mental health centre and traditional day centres.	Open meetings are held twice a week in which service users and staff discuss and organise the management of the centre.	Difference in hospitalisation rates and use of psychiatric medication. Focus group quotes.
Palmer et al.^19^	Australia	Randomised controlled trial, cluster design involving four mental health organisations, patient involvement implemented in nine teams.	Psychosocial recovery‐oriented Mental Health Community Support Services (MHCSS).	People with long‐term psychosocial impairments because of mental illness.	Investigate the impact of experience‐based co‐design (EBCD) of mental health services on psychosocial recovery.	EBCD involves interviews/focus groups with service users. Collaboration in determining priorities and action plans.	Questionnaire (Revised recovery assessment scale—RASR) to measure psychosocial recovery Quality of life (UROHIS‐QoL eight‐item index).
Usman et al.^20^	Ireland	Mixed methods questionnaire.	The Psychiatry of Later Life (POLL) community‐based service.	Aged >65 years with mental health difficulties and dementia patients with behavioural disturbance or psychiatric symptoms.	To obtain the views of service users regarding the service and address issues identified.	Audit of service users' views via questionnaire. A cycle of improvements is done after each audit.	Patient satisfaction and user comments regarding service.
Livingston et al.^21^	Canada	Mixed methods study. Quantitative and qualitative data were gathered from service users twice during the 19‐month patient involvement scheme.	Forensic mental health hospital.	Forensic mental health hospital inpatients.	To increase patient engagement by strengthening a patient advisory committee (PAC) and determine what effects this has on perceived service improvements.	PAC: Monthly meetings of patients and staff to discuss hospital‐wide issues and concerns.	Improvements in patient engagement, valuing patient preferences and service user comments.
Wang et al.^22^	Canada	Quantitative descriptive study—data collected by clinicians (including questionnaires) extracted from clinic databases.	Youth Wellness Centre (YWC) provides mental health and addiction services.	Emerging adults aged 17–25.	To evaluate how well the YWC is serving populations.	Preferences of service users collected and incorporated into service design. Implementing a youth council to maintain ongoing involvement.	Number of new clients, satisfaction scores, attendance rates and referral methods.
Lwembe et al.^23^	United Kingdom	Qualitative study, interviews and focus groups.	Improving access to psychological therapies (IAPT) service.	Black and minority ethnic (BME) communities.	Evaluate a pilot‐coproduced mental health IAPT service to meet the needs of the BME communities.	Staff and patients co‐designed and co‐delivered IAPT services to BME communities using the principles of co‐production	Treatment completion rate and interview quotes.
Springham and Robert^24^	United Kingdom	Quantitative descriptive.	Hospital acute mental health triage ward.	Ward patients	To see if using EBCD to redesign procedures and address issues would reduce formal complaints.	Gather patient experiences through narrative‐based interviews. Staff and patients identify priorities and co‐design solutions.	Formal complaints regarding the ward.
Parkes et al.^25^	United Kingdom	Qualitative study interviews—two phases—one before and one after the new service is introduced.	Psychiatric wards	People with learning disabilities require an acute psychiatric admission.	To incorporate the views of service users in the development of an integrated psychiatric service for people with learning disabilities.	Interviews with service users.	Qualitative quotes.
Illback and colleagues^15^, ^16^, ^17^	Ireland	Quantitative descriptive.	Jigsaw: Provides early access care for young people with mild‐to‐moderate issues with mental health. Aims to fill the gap and ensure continuity of care for youth.	Young people aged 12–25.	To make current services more accessible by engaging communities in planning, design and implementation. Moreover, to determine whether this reduces psychological distress.	Service planning team meetings consisted of young people and staff. Focus groups with young people. Implementation of a youth advisory panel to inform service delivery.	Psychological distress, referral methods and client demographics.

Methods of service user involvement (inputs) varied between studies and are described below. As expected from the broad eligibility criteria, a range of outputs were identified and extracted. Quantitative outputs included hospitalisation rates, medication use, psychosocial recovery, satisfaction scores, service attendance, formal complaints, treatment completion and client demographics. Qualitative outputs took the form of service user views obtained from interviews and focus groups.

### 3.3 Quality assessment

The overall quality of the included studies was good (Supporting Information: FS4). All studies involved a clear research question and appropriate data collection methods to answer them. However, there was some risk of bias in regard to missing data^22^ and sample generalisability,^18^, ^21^ Furthermore, one study was prone to selection bias due to the use of postal questionnaires^20^ and another study was prone to participant bias due to self‐reported hospitalisation rates.^18^ Effect sizes were only reported in three of the nine studies,^18^, ^19^, ^22^ thus limiting interpretation on whether findings had any practical significance.

### 3.4 Study results

Results were mapped onto a logic model (Table 2) to show methods of involvement, service changes and resulting outputs. Working together with a lived experience advisory panel (Supporting Information: FS7), each study was characterised by its level of involvement based on the ladder of co‐production.^2^

**Table 2:** Logic model of involvement methods, activities and outputs.

**Author/Year**	**Level of involvement**	**Service type**	**Inputs (Method of involvement)**	**Activities**	**Outputs**
Pocobello 2020	Co‐production	Co‐produced community mental health day centre which organises activities, support groups etc	Involvement of users at every stage including service evaluation. Ongoing involvement as assemblies held twice weekly and service users involved in running of the service. Power imbalances are mitigated by having a user majority in assemblies and ensuring it is the main place where decisions are made.	– No regulation of how users' time is spent/free agency. – Openness of centre, users can go where they want and when they want. – Continuity of therapeutic relations and access to a social network – Users can work for the centre ‐ become involved. – Opportunity for users to take on responsibilities to become self‐confident.	– Increased engagement with centre compared to traditional services – evidenced by usage statistics. – 63% fewer lifetime mental health related hospitalisations reported when compared with traditional services (*p* = 0.002). – 39% users reported reduction/withdrawal from psychiatric medication since using service in contrast to 22% in traditional services (*p* = 0.022). – Feeling respected and treated as equal. – Feeling empowered towards recovery. – Reduced distinction between users and staff.
Lwembe 2017		Improving access to psychological therapies (IAPT) service	Using the principles of co‐production, mental health practitioners and service users co‐designed and co‐delivered IAPT services. This included engaging and training local residents as mental health champions. Aims of the service were collectively agreed upon by all stakeholders. Users had decision‐making power regarding content of psychological sessions.	– Service delivered at a community setting. – Employing an expert patient from the BME community to co‐deliver psychological interventions. – Changing service name to acknowledge stigma of mental health. – Adopted a holistic approach by addressing other issues patients face e.g. debt, childcare.	– Increased treatment accessibility for BME users. – Increased motivation of patients to attend and complete treatment (73% completion in pilot). – Patients felt their needs being met due to holistic approach – Increased service uptake over 6 weeks.
O'Reilly 2022 O'Keeffe 2015 Illback 2010	**Co‐design**	Jigsaw: provides early access care for young people with mild – moderate issues with mental health. Aims to fill the gap and ensure continuity of care for youth.	Service users were part of planning team meetings, and helped to prioritise key issues, assess resources required and determine measurable goals. Focus groups with vulnerable communities used to discuss service needs and changes. Implemented youth advisory panel (YAP) made up of young people who can inform service practice.	– Adopted a centre‐based drop in approach. – Physical environment designed by young people. – Increased technology use e.g. social media to increase help—seeking knowledge. – No professional referral required. – Holistic approach offering consultations with parents/teachers on mental health support. – Addressing continuation of care issues when referred to secondary services. – Providing early access care for mild mental health issues.	– Significant reduction in psychological distress after engaging in service. – Increased male engagement assessed via service usage statistics. – Increased accessibility (87% of referrals not from health professionals). – Increasing number of referrals each year.
Wang 2019		Youth Wellness Centre (YWC) providing mental health and addiction services	Preferences of service users (youth) collected quantitatively. Youth involvement in design meetings and ongoing involvement via the youth council. Service user consultations throughout development phase.	– Location of service in central downtown location. – A mobile team to target hard‐to‐reach populations. – Addressing transition from child to adult services. – Implementing an early intervention service for youth with mild mental health concerns.	– New clients have increased by 20% in first 3 years. – Satisfaction scores regarding service quality are higher than provincial mean (*d* = 0.36 and *d* = 0.28). – High attendance of orientation (90.9%) and initial assessments (87.2%). – 46.5% of users self‐referred.
Palmer 2021		Psychosocial recovery‐oriented Mental Health Community Support Services (MHCSS)	Interviews to identify touch points and focus groups using emotional mapping to understand experiences. Determine objectives and create process maps in co‐design meetings.	– Prioritising continuity of care via secondary worker process for staff leave – Introduced newsletter and calendar of social events, – Outreach policies and voicemail system updated. – Implemented feedback system, box near reception. – Redesigned reception to make it more welcoming. – Implemented carer peer support workers within services.	No significant differences found in either psychosocial recovery or quality of life between co‐designed or control services.
Springham 2015		Hospital acute mental health triage ward	Gathered patient/carer experiences via observation and 15 interviews. Established a network of service users to help identify priorities for change and co‐design solutions to issues.	– No longer applying blanket rules such as removing personal items. – Prioritised communication on admission. – Abandoned triage model as two‐stage process so patients would not have to communicate with two sets of staff.	– 23 continuous months with no formal complaints, lower than neighbouring wards.
Livingston 2013	**Engagement**	Forensic mental health hospital	Patient representatives and management staff met via a patient advisory committee (PAC) monthly to discuss service concerns.	– Reinstated availability of caffeinated coffee for patients. – Extended evening curfew on weekends and holidays.	– 45.8% of patients stated that they had seen “moderate” or “extreme” improvements in the service. – Majority (57.1%) of surveyed staff stated that patients' experiences had been “moderately” or “extremely” improved.
Parkes 2007	**Consultation**	Psychiatric wards	Semi‐structured interviews of service users with learning disabilities regarding their views. Information incorporated into service development process.	– More emphasis on orientating/supporting patients on admission. – Employing a dedicated pharmacist to explain medication to patients. – Prioritising making ward rounds less intimidating	– Majority of patients reported settling in quickly and not feeling frightened. – Greater understanding of medication prescribed. – View of ward rounds remained negative.
Usman 2021		The Psychiatry of Later Life (POLL) community‐based service	Questionnaire sent to service users from which improvements to service were made. Questionnaire sent 4 times over 8 years.	– Improved access to disabled parking spaces. – Home visits offered to those with significant physical health issues. – Leaflet about the discharge and re‐referral process introduced. – Implement a carer support group.	– >90% satisfied with waiting time and information provided. – 85% of comments in the questionnaire were positive, mainly regarding supportive staff. – 91.2% very satisfied or satisfied with support in recovery.

#### 3.4.1 Involvement methods

**Figure 3:**
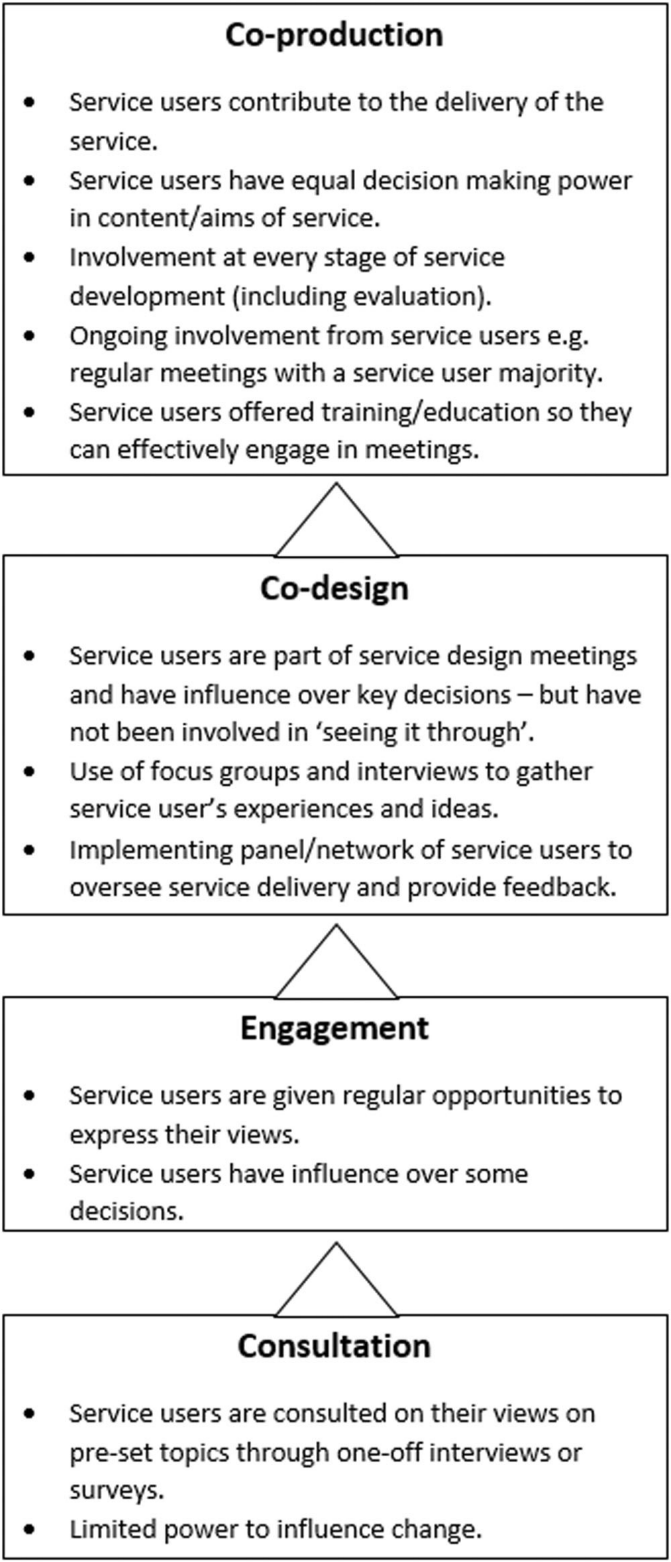
Involvement methods used in included studies, matched against the ladder of co‐production.^2^

Brief summaries of the methods used in each paper and where they fit on the ladder of the co‐production are shown in Figure 3. Of the nine studies, two were characterised as co‐production.^18^, ^23^ Both studies emphasised the importance of sharing power between staff and service users, a practice that is fundamental to co‐production. Co‐production requires working with service users from design to delivery as exemplified by Pocobello et al.,^18^ who reported involvement at every stage, including service evaluation. Service users worked alongside staff to progress service design at open assembly meetings and through employment opportunities to implement changes and co‐deliver services.^18^ The principles of the service emphasised the importance of equal partnership between staff and service users in managing the service.^18^ Power imbalances were mitigated by ensuring the weekly assemblies always had a service user majority, and professionals avoided using jargon and shared their knowledge in accessible ways.^18^ Decisions about the service were made at these assembly meetings.^18^ Similarly, Lwembe et al.,^23^ reported using co‐production principles when bringing together service users and staff to co‐design and co‐deliver ‘improving access to psychological therapies’ services. Service users had the opportunity to become mental health champions, involving targeted outreach in the community, and to co‐deliver psychological interventions to enhance physical activity.^23^ Service users were also instrumental in strategic decision‐making, for example, changing the service name to ‘A Step to Liveliness’ to reduce stigma.^23^

Four studies were classified as using co‐design approaches to involvement.^15^, ^16^, ^17^, ^19^, ^22^, ^24^ Although service users in these studies influenced service design, they were not involved in delivery and lacked equal power. The Jigsaw study reported a service that was codesigned by including service users in planning meetings and creating a youth advisory panel to provide feedback.^15^, ^16^, ^17^ They used service user focus groups, including those from vulnerable communities, to identify their needs from a new service.^15^, ^16^, ^17^ However, equal power sharing was not present in aspects of the co‐design process, and focus groups were led by staff. Service users were not involved in service delivery.^15^, ^16^, ^17^ A similar process was used by Wang et al., who also reported a new youth mental health service that involved service users in design meetings. However, the power to make changes remained with professionals.^22^ Similarly, Palmer et al.^19^ and Springham and Robert^24^ described co‐design processes in which service users were instrumental in prioritising service objectives and formulating design plans in working groups. Palmer et al. partially addressed power imbalances by providing training in effective engagement in co‐design meetings. This was not considered to be co‐production as professionals led the co‐design process and the final implementation of change.^19^

The involvement described by Livingston et al.^21^ represents the engagement of service users. In contrast to simple consultation, service users had enhanced opportunities to express their views; however, they had limited capacity to influence change.^2^ This was exemplified by the use of a patient advisory committee (PAC) that comprises service users and staff to discuss service issues.^21^

Service user involvement was limited to consultation in the remaining two studies, in which service users were either asked about their views through a semistructured interview with a professional^25^ or a questionnaire asking about their experiences.^20^ These consultation methods did not give service users the power to influence change.

#### 3.4.2 Activities

A range of activities were reported as shown in the logic model (Table 2). These included prioritising continuation of care,^15^, ^16^, ^17^, ^18^, ^19^, ^22^ employment of service users,^18^, ^23^ adoption of a holistic approach to mental health,^15^, ^16^, ^17^, ^18^, ^23^ redesigning the physical environment,^15^, ^16^, ^17^, ^19^ influencing service location^22^, ^23^ and changing admission processes.^24^, ^25^ Generally, service changes reported by studies in which involvement was limited to consultation or engagement were mostly quick fixes and process oriented, for example, disabled parking and providing coffee. Studies that emphasised involvement through co‐design or co‐production reported changes that were more fundamental, for example, adopting a holistic approach, employment of service users and novel types of services.

#### 3.4.3 Outputs

Increased service attendance and treatment completion were reported by four studies, all of which utilised co‐production or co‐design methods.^15^, ^16^, ^17^, ^18^, ^22^ Three of these studies also reported increased accessibility of services, including increased self‐referral.^15^, ^16^, ^17^, ^22^, ^23^

Improvements in service user mental health outputs were reported by two studies in which services were rated as co‐produced or co‐designed.^15^, ^16^, ^17^, ^18^ Pocobello et al.^18^ evaluated a co‐produced day centre for people with (unspecified) mental health difficulties (*n* = 37), by using a questionnaire to ask how many times they had been hospitalised for mental health reasons since frequenting the centre.^18^ They then compared the responses to those of service users at three traditional (not co‐produced) day centres in the same region (*n* = 40).^18^ After adjusting for confounders between groups, they reported that those using the co‐produced service had 63% fewer hospitalisations since attending the centre when compared to users of the comparator (traditional) services (*p* = .002).^18^ However, due to the nonrandomised design, the reasons for this difference are unclear. In addition, 39% of service users reported reduction or withdrawal from psychiatric medication since using the new service, compared with 22% among those using traditional services (*p* = .022).^18^ Reduction in psychiatric medication use was identified by service users in this study as an indicator of successful co‐production.^18^

Users of the co‐designed Jigsaw service had their psychological distress measured via questionnaires during their first and final sessions.^16^ Results showed that 62% of service users experienced a significant improvement in psychological distress scores, although the lack of a control group limits the interpretation of these findings.^16^ By contrast, another study found no significant differences in self‐reported psychosocial recovery after co‐designed changes were implemented in the service.^19^ Three of the studies evaluating services rated as co‐design^22^, ^24^ and consultation^20^ reported improvements in patient satisfaction via questionnaires^20^, ^22^ or reduction in formal complaints.^24^ However, only one of these studies found a statistically significant improvement in patient satisfaction.^22^ This was indicated by two out of three items on a satisfaction questionnaire scoring significantly higher than the provincial average (*d* = 0.36 and *d* = 0.28).^22^

Discussion with our lived experience advisory panel highlighted differences between professionals and service users in the importance attached to different outputs. For example, an improved perception of services may be valued more highly by service users, whereas professionals may attach more value to treatment completion rates.

#### 3.4.4 Links between inputs, activities and outputs

As part of the process of creating our logic model (Table 2), we sought to elucidate links between involvement methods, service changes and reported outputs. Users of the co‐produced day centre described by Pocobello et al. reported feeling respected due to the reduced distinction between users and professionals and the emphasis on working together. This study demonstrated an association between co‐production principles and positive relationships between service users and staff^18^:

citizens who are here to do something together (…) rather than finding yourself closed in a room with a specialist, with a psychiatrist or a psychologist seated behind a table who poses questions (.) who exploits and judges us from above…^18^ ^,p.469^

This service also empowered service users to achieve recovery by focusing on social inclusion in addition to medication, and by giving users the freedom to organise their time, in contrast to traditional services where they may feel passive.^18^ This recovery was evidenced by the lower (self‐reported) hospitalisation rates and medication need when compared to traditional services.^18^ Service changes such as removing time regulations, adopting an open door policy and ensuring continuity of therapeutic relations were made as a result of service user input within assemblies, and mitigation of power imbalances between users and professionals.^18^ The involvement of service users in the day‐to‐day management of the service played a key role in improving mental health outputs and contributed to the positive outlook reported by users.^18^ This is highlighted in the following patient quote:

We start wishing to improve our mental health (.) not only based on medication but with the activities we are doing in the centre, and, overall, with the fact that we are taking responsibilities, being more self‐confident…I never experienced something like this before.^18^ ^,p.471^

Lwembe et al.^23^ also employed service users as expert patients as part of their co‐produced approach. Interview quotes from this study highlight the comfort this provided to service users as well as the rise in cultural competence which was cited as important in disclosing and hearing personal experiences and ensuring attendance.^23^ Descriptions of comfort, respect and safety reported by service users were more common in studies that reported substantial involvement.^18^, ^23^

This study also highlighted the importance of service user involvement in increasing service uptake by addressing distrust of mental health services and ensuring transparency and cultural competence in the new service.^23^ The reported increase in service uptake was also attributed to the community location of the service, which was proposed and agreed upon by service users, allowing for face‐to‐face appointments with those who cannot travel.^23^ Furthermore, as described in other studies,^15^, ^16^, ^17^, ^18^, ^19^, ^22^ involving service users ensured that a holistic approach was taken by encouraging services to consider service users' social and financial concerns.^23^

Both the Jigsaw service^15^, ^16^, ^17^ and Youth Wellness Centre (YWC)^22^ co‐designed services for young people and found that demand for these services increased.^15^, ^16^, ^17^, ^22^ Both services removed the requirement for professional referral, after which 87% of Jigsaw referrals and 47% of YWC referrals came from nonprofessional sources, including self‐referral.^15^, ^16^, ^17^, ^22^ The Jigsaw service also found an increase in male engagement, suggesting that young men were more comfortable self‐referring rather than accessing help via more formal referral pathways.^15^, ^16^, ^17^

Springham and Robert^24^ also showed how service users may prioritise different issues to staff when developing services. For example, whereas staff prioritised key procedures during admissions, for example, care plans and medication, service users prioritised communication to allay anxiety among those newly admitted.^24^ Service user feedback was vital in restoring the relational aspect of mental health care, which in turn was associated with an absence of formal complaints over 23 months.^24^ When complaints subsequently rose again, the authors reflected on the importance of ongoing collaboration between service users and staff to maintain the benefits of involvement.^24^

Parkes et al.^25^ consulted with service users but reported that ward rounds continued to be viewed negatively by service users, despite work to improve them. This could be indicative of the involvement methods used. Interviews allowed users to describe their experiences of the service but did not provide opportunities to offer solutions or be involved in service design.^25^ This supports the view that lesser forms of involvement may limit the outputs that can be achieved.

## 4 DISCUSSION

### 4.1 Main findings

We aimed to understand how service user involvement influences service commissioning, development and delivery, and to consider if/how this leads to service level and service user outcomes. We set out to test the hypothesis that involvement at or near the top of the co‐production ladder leads to better outcomes than lesser forms of involvement.

We identified nine studies that described service user involvement and service‐level outputs, ranging from consultation to co‐production. Although evidence of early change (outputs) was found, longer term outcomes were rarely reported. A logic model approach was used to establish potential causal links between inputs (involvement methods) and outputs. Included studies reported a spectrum of involvement methods which were characterised according to the ladder of co‐production.^2^ Studies implementing co‐production^18^, ^23^ and co‐design^15^, ^16^, ^17^, ^19^, ^22^, ^24^ described patient involvement throughout all stages of the development process, with co‐produced studies highlighting the importance of sharing power between service user and staff. In contrast, studies characterised as engagement^21^ or consultation^20^, ^25^ described limited patient capacity to influence change.

The results showed a mixed picture regarding outputs of involvement, with most reporting involvement to be associated with positive outputs such as increased patient satisfaction. However, studies reporting more extensive involvement found more substantial effects on service organisation and delivery. Implementing co‐production or co‐design methods led to more activities targeted at structural and cultural aspects of the service. As a result, these studies reported service‐level outputs that may be valued more by service commissioners, for example, increased treatment completion rates,^22^, ^23^ increased referrals,^15^, ^16^, ^17^, ^23^ improved patient mental health outcomes^16^, ^18^ and improved treatment accessibility.^15^, ^16^, ^17^, ^23^ In contrast, lesser forms of involvement such as consultation and engagement approaches were mostly limited to activities regarding environmental changes such as car‐parking accessibility and in‐patient curfews.^20^, ^21^ This in turn led to more limited outputs such as improved perception of services.^20^, ^21^ Therefore, findings suggest that outputs related to service effectiveness are achieved by more involved approaches such as co‐production.

### 4.2 Strengths and limitations

Previous research has investigated the effects of service‐user involvement on those who were actually involved in the process.^6^, ^7^, ^9^ A strength of this review is its focus on outputs assessed at the service level, rather than according to the views of those who took part in service development. This review addresses the gap identified by Crawford et al.,^7^ by examining the changes in the quality of services associated with greater or lesser service user involvement.

Presenting findings in the form of a logic model allows for a comparison of involvement methods, the associated outputs and whether lesser forms of involvement are limited in their outputs. This research supports the value of co‐production in mental health services, which may guide future service planning and best practice regarding patient involvement. A further strength of this review is the involvement and contribution of a lived experience panel in analysing the results. The panel highlighted the potential divergence in how certain outputs may be valued between service users and professionals. Therefore, when evaluating the impact of service user involvement, it is important to consider what outputs are captured and how this may affect the interpretation of the effects of service user involvement. Long‐term service outcomes were rarely reported by studies, thus limiting the scope of this review in addressing whether involvement leads to long‐term service impacts.

Despite the associations between service effectiveness and co‐production, causality is difficult to establish from a small sample of complex services, which used diverse involvement methods. Although the sample heterogeneity allowed for the comparison of involvement methods within different contexts, this variation may also limit findings due to potential confounders within studies. For example, co‐design methods in a community centre may differ from those in a hospital ward. Furthermore, different outputs were measured for each study, making comparison difficult. Therefore, these inferences should be viewed as preliminary findings. Additionally, studies were often uncontrolled in design, with the exception of Pocobello et al.^18^ and Palmer et al.^19^ It was possible that inferences based on these comparisons were biased as a result of the nonrandomised, unblinded nature of these studies, or confounded by context or type of service being evaluated. Furthermore, most studies included in this review lacked information about the nature or severity of mental health problems for which help was sought, which may impact service development and outcomes.^18^, ^21^, ^22^, ^23^, ^24^ Findings must also be interpreted with caution as some methods of output measurements were prone to chance (arising from small sample sizes), reporting, recall and selection bias and confounding.^19^, ^22^

As stated by previous reviews,^7^, ^9^, ^10^, ^12^, ^26^ there is a lack of rigorous evaluation of patient involvement and associated outputs, as it is difficult to separate context from impact and ensure no other factors are contributing to measured outputs. More studies should therefore adopt a design in which service outputs are compared to services which do not incorporate patient involvement to further demonstrate the benefits of patient involvement and allow recommendations on best practices.

## 5 CONCLUSIONS

Co‐production and co‐design were associated with more service and patient‐level outputs than more limited forms of involvement such as service user consultation. The mechanisms that contribute to these outputs may include continuation of care, treatment accessibility and increased alignment of services to patient needs. Limited forms of involvement were associated with service user perceptions rather than more objective measures of change. However, it is important to consider how these outputs may be equally important as service‐level indicators such as attendance rates. As highlighted by our lived experience panel, improved perception of services may be valued more highly by service users, whereas professionals may attach more value to clinical outcomes. Therefore, highlighting a need to improve both clinical and patient experience outcomes to ensure an effective service is used by the patients it targets. The findings of this review may contribute to future service planning by motivating more involvement forms of service user participation in service development and delivery.

## AUTHOR CONTRIBUTIONS

Naseeb Ezaydi was involved in searches, screening, data extraction, analysis, interpretation and drafting of this article. Elena Sheldon was involved in the screening, data extraction, interpretation and drafting of this article. Scott Weich, Alex Kenny and Elizabeth Taylor Buck were involved in the interpretation and drafting of this article.

## CONFLICT OF INTEREST STATEMENT

The authors declare no conflict of interest.

## Supporting information

Supporting information.

Supporting information.

Supporting information.

Supporting information.

Supporting information.

Supporting information.

Supporting information.

## Data Availability

Data sharing is not applicable to this article as no new data were created or analysed in this study. The full search strategy and data extraction form have been included in the Supporting Information. Any other supporting data is available from the authors on request.
