# Supplementary material for: Service user involvement in mental health service commissioning, development and delivery: A systematic review of service level outcomes
Source: Health Expect. 2023 Jun 8;26(4):1453–66. doi: 10.1111/hex.13788 (PMC10349231; doi:10.1111/hex.13788)
Supplement: Supplementary file 2 — Supporting information. [file HEX-26--s001.docx]

***Supporting Information 2:*** *Search strategies.*

Total hits: **10,899**

After duplicate removal: **9,907**

**Embase Search**

1996 to 2022 Week 47

1 exp mental health/ 200378

2 exp psychiatry/ 112121

3 exp psychology/ 359774

4 1 or 2 or 3 633449

5 service.mp. 786114

6 patient.tw. 3573218

7 client.tw. 36170

8 service user.tw. 2475

9 5 or 6 or 7 or 8 4218214

10 4 and 9 146523

11 co-production.mp. 2669

12 coproduction.tw. 670

13 co-design.mp. 2082

14 co-creation.tw. 951

15 cocreation.tw. 138

16 experience based design.mp. 35

17 involv*.tw. 2865011

18 collaborat*.tw. 247440

19 engag*.tw. 285728

20 participa*.tw. 1942457

21 codesign.tw. 354

22 11 or 12 or 13 or 14 or 15 or 16 or 17 or 18 or 19 or 20 or 21 4884782

23 design.tw. 1662187

24 commission*.tw. 53454

25 deliver*.tw. 972264

26 develop*.tw. 5803075

27 manage*.tw. 1963562

28 23 or 24 or 25 or 26 or 27 8938098

29 22 and 28 1980327

30 service.mp. 786114

31 outcomes.mp. 1993218

32 changes.mp. 2442552

33 improv*.mp. 3990656

34 31 or 32 or 33 7220101

35 30 and 34 268080

36 attendance.mp. 43486

37 accessibility.mp. 64216

38 satisfaction.mp. 321610

39 recovery.mp. 608535

40 waiting time.mp. 12147

41 35 or 36 or 37 or 38 or 39 or 40 1259011

42 10 and 29 and 41 12638

43 limit 42 to (english language and embase and english and article and journal) 3561

**3561 hits**

**3488 hits** after duplication removal

**Saved for abstract screening:**

**Saved for full text screening:**

**Included:**

**MEDLINE Search**

Ovid MEDLINE(R) and Epub Ahead of Print, In-Process, In-Data-Review & Other Non-Indexed Citations and Daily <1946 to June 13, 2022>

1 exp Mental Health/ 52179

2 exp Psychiatry/ 108361

3 exp Psychology/ 69783

4 1 or 2 or 3 222549

5 service.mp. 416939

6 patient.tw. 2497063

7 client.tw. 31193

8 service user.tw. 1713

9 5 or 6 or 7 or 8 2852963

10 4 and 9 27132

11 co-production.mp. 2139

12 coproduction.tw. 622

13 co-design.mp. 1371

14 codesign.tw. 250

15 co-creation.tw. 667

16 cocreation.tw. 123

17 experience based design.mp. 22

18 involv*.tw. 2513292

19 collaborat*.tw. 174942

20 engag*.tw. 220767

21 participa*.tw. 1466049

22 11 or 12 or 13 or 14 or 15 or 16 or 17 or 18 or 19 or 20 or 21 4042496

23 design.tw. 1349769

24 commission*.tw. 46150

25 deliver*.tw. 749217

26 develop*.tw. 4936532

27 manage*.tw. 1497882

28 23 or 24 or 25 or 26 or 27 7481000

29 22 and 28 1488582

30 service.mp. 416939

31 outcomes.mp. 1225380

32 changes.mp. 2305008

33 improv*.mp. 2966127

34 31 or 32 or 33 5731035

35 30 and 34 132100

36 attendance.mp. 29954

37 accessibility.mp. 132183

38 satisfaction.mp. 233747

39 recovery.mp. 544659

40 waiting time.mp. 7835

41 35 or 36 or 37 or 38 or 39 or 40 1035639

42 10 and 29 and 41 1661

**1661 hits**

**Saved for abstract screening:** 40

**Saved for full text screening:** 8

**Included:** 5

**PsychInfo Search**

**APA PsycInfo <1806 to June Week 4 2022>**

1 exp Mental Health/ 79404

2 exp Psychiatry/ 54770

3 exp Psychology/ 224031

4 1 or 2 or 3 344641

5 service.mp. 176869

6 patient.tw. 269790

7 client.tw. 55784

8 service user.tw. 2306

9 5 or 6 or 7 or 8 475440

10 4 and 9 41551

11 co-production.mp. 680

12 coproduction.tw. 272

13 co-design.mp. 488

14 codesign.tw. 90

15 co-creation.tw. 1484

16 cocreation.tw. 245

17 experience based design.mp. 7

18 involv*.tw. 505228

19 collaborat*.tw. 96334

20 engag*.tw. 231002

21 participa*.tw. 805645

22 11 or 12 or 13 or 14 or 15 or 16 or 17 or 18 or 19 or 20 or 21 1396983

23 design.tw. 319075

24 commission*.tw. 14021

25 deliver*.tw. 119222

26 develop*.tw. 1227369

27 manage*.tw. 321224

28 23 or 24 or 25 or 26 or 27 1708746

29 22 and 28 568887

30 service.mp. 176869

31 outcomes.mp. 337902

32 changes.mp. 393505

33 improv*.mp. 510684

34 31 or 32 or 33 1072764

35 30 and 34 54768

36 attendance.mp. 21480

37 accessibility.mp. 25323

38 satisfaction.mp. 149334

39 recovery.mp. 78931

40 waiting time.mp. 997

41 35 or 36 or 37 or 38 or 39 or 40 314039

42 10 and 29 and 41 2623

**2623 hits**

**Saved for abstract screening:** 35

**Saved for full text screening:** 5

**Included:** 1

**CINAHL Search**

**3054 hits**

**Saved for abstract screening:** 46

**Saved for full text screening:** 11

**Included:** 5 (studies: 3)

| **#** | **Query** | **Limiters/Expanders** | **Last Run Via** | **Results** |
| --- | --- | --- | --- | --- |
| S16 | S3 AND S6 AND S11 | Limiters - Abstract Available  Expanders - Apply equivalent subjects  Narrow by SubjectMajor: - community health services  Narrow by SubjectMajor: - health services needs and demand  Narrow by SubjectMajor: - patient satisfaction  Narrow by SubjectMajor: - program evaluation  Narrow by SubjectMajor: - collaboration  Narrow by SubjectMajor: - consumer participation  Narrow by SubjectMajor: - health services accessibility  Narrow by SubjectMajor: - community mental health services  Narrow by SubjectMajor: - mental health services  Search modes - Boolean/Phrase | Interface - EBSCOhost Research Databases  Search Screen - Advanced Search  Database - CINAHL | 3,054 |
| S15 | S3 AND S6 AND S11 | Limiters - Abstract Available  Expanders - Apply equivalent subjects  Search modes - Boolean/Phrase | Interface - EBSCOhost Research Databases  Search Screen - Advanced Search  Database - CINAHL | 7,981 |
| S14 | S3 AND S6 AND S11 | Limiters - Linked Full Text; Abstract Available  Expanders - Apply equivalent subjects  Search modes - Boolean/Phrase | Interface - EBSCOhost Research Databases  Search Screen - Advanced Search  Database - CINAHL | 1,542 |
| S13 | S3 AND S6 AND S11 | Limiters - Abstract Available  Expanders - Apply equivalent subjects  Search modes - Boolean/Phrase | Interface - EBSCOhost Research Databases  Search Screen - Advanced Search  Database - CINAHL | 8,297 |
| S12 | S3 AND S6 AND S11 | Expanders - Apply equivalent subjects  Search modes - Boolean/Phrase | Interface - EBSCOhost Research Databases  Search Screen - Advanced Search  Database - CINAHL | 8,297 |
| S11 | S9 OR S10 | Expanders - Apply equivalent subjects  Search modes - Boolean/Phrase | Interface - EBSCOhost Research Databases  Search Screen - Advanced Search  Database - CINAHL | 565,172 |
| S10 | attendance OR accessibility OR satisfaction OR recovery OR waiting time | Expanders - Apply equivalent subjects  Search modes - Boolean/Phrase | Interface - EBSCOhost Research Databases  Search Screen - Advanced Search  Database - CINAHL | 389,281 |
| S9 | S7 AND S8 | Expanders - Apply equivalent subjects  Search modes - Boolean/Phrase | Interface - EBSCOhost Research Databases  Search Screen - Advanced Search  Database - CINAHL | 233,413 |
| S8 | service | Expanders - Apply equivalent subjects  Search modes - Boolean/Phrase | Interface - EBSCOhost Research Databases  Search Screen - Advanced Search  Database - CINAHL | 687,625 |
| S7 | outcomes OR changes OR improv* | Expanders - Apply equivalent subjects  Search modes - Boolean/Phrase | Interface - EBSCOhost Research Databases  Search Screen - Advanced Search  Database - CINAHL | 2,007,554 |
| S6 | S4 AND S5 | Expanders - Apply equivalent subjects  Search modes - Boolean/Phrase | Interface - EBSCOhost Research Databases  Search Screen - Advanced Search  Database - CINAHL | 365,454 |
| S5 | design OR commission OR deliver OR develop OR manage OR improve | Expanders - Apply equivalent subjects  Search modes - Boolean/Phrase | Interface - EBSCOhost Research Databases  Search Screen - Advanced Search  Database - CINAHL | 1,311,044 |
| S4 | coproduction OR ( co-production or collaboration or collaborative or co-design ) OR ( codesign or co-design ) OR involv* OR collaborat* OR ( cocreation or co-creation ) OR experience-based design OR participa* OR engag* | Expanders - Apply equivalent subjects  Search modes - Boolean/Phrase | Interface - EBSCOhost Research Databases  Search Screen - Advanced Search  Database - CINAHL | 1,108,104 |
| S3 | S1 AND S2 | Expanders - Apply equivalent subjects  Search modes - Boolean/Phrase | Interface - EBSCOhost Research Databases  Search Screen - Advanced Search  Database - CINAHL | 99,854 |
| S2 | service OR patient OR client OR service user | Expanders - Apply equivalent subjects  Search modes - Boolean/Phrase | Interface - EBSCOhost Research Databases  Search Screen - Advanced Search  Database - CINAHL | 2,834,522 |
| S1 | mental health OR psychiatry OR psychology | Limiters - Abstract Available; English Language  Expanders - Apply related words; Apply equivalent subjects  Search modes - Boolean/Phrase | Interface - EBSCOhost Research Databases  Search Screen - Advanced Search  Database - CINAHL | 184,518 |
