# Supplementary material for: Service user involvement in mental health service commissioning, development and delivery: A systematic review of service level outcomes
Source: Health Expect. 2023 Jun 8;26(4):1453–66. doi: 10.1111/hex.13788 (PMC10349231; doi:10.1111/hex.13788)
Supplement: Supplementary file 3 — Supporting information. [file HEX-26--s003.docx]

***Supporting information 3:*** *Inclusion and exclusion criteria*

| **Selection Criteria** | **Inclusion** | **Exclusion** | **Justification** |
| --- | --- | --- | --- |
| Population | Mental health services | Staff training, development of tools, research interventions, guidelines | Only services will be included to ensure the research question is answered regarding service commissioning, development and delivery. |
| Intervention | Any form of patient involvement that describes participation in the commissioning, design, monitoring, development and delivery of mental health services.  In mental health service settings, including acute healthcare and community settings. | This will not include patient involvement in research or shared management of individual treatment choices within patient-provider consultations. | Patient involvement in research is mainly focused on improving the quality of research rather than improving services.  Involvement in individual treatment plans does not constitute service improvements as it only affects the individual. |
| Comparators | Usual engagement / no involvement/ uncontrolled. |  | Broad eligibility framework to allow for anticipated scarcity of studies |
| Outcomes | Direct patient-focused reported changes in mental health services associated with involvement as reported by patients, caregivers or health service providers. Outcomes may be reported both quantitatively or qualitatively.  These may include changes in service structure, attendance, accessibility, patient reported satisfaction or health indicators. | Outcomes regarding patient or staff perspectives on the involvement process will not be included. | Only outcomes related to changes in the service as a result of patient involvement will be included as outcomes related to the involvement process do not affect all service users. |
| Study type | No restrictions on study designs. Eligible studies will include those that are observational, service evaluations, or controlled trials.  Published in peer-reviewed journals to ensure reliability of results. |  | Broad eligibility framework to allow for anticipated scarcity of studies |
| Language | English |  | Only studies published in English will be included due to limited translation resources. |
| Other | No restrictions on the date of publication. |  | Broad eligibility framework to allow for anticipated scarcity of studies |
