# Supplementary material for: Service user involvement in mental health service commissioning, development and delivery: A systematic review of service level outcomes
Source: Health Expect. 2023 Jun 8;26(4):1453–66. doi: 10.1111/hex.13788 (PMC10349231; doi:10.1111/hex.13788)
Supplement: Supplementary file 4 — Supporting information. [file HEX-26--s004.docx]

***Supporting Information 4:*** *Quality assessment results - table adapted from MMAT (2018).*

| **Type of study** | **Author/ year** | **Screening questions** | | **Methodological quality criteria** | | | | |
| --- | --- | --- | --- | --- | --- | --- | --- | --- |
| **Qualitative**  **(ref.)** |  | Are there clear research questions? | Does the collected data address the research questions? | Is the qualitative approach appropriate to answer the research question? | Are the qualitative data collection methods adequate to address the research question? | Are the findings adequately derived from the data? | Is the interpretation of results sufficiently substantiated by data? | Is there coherence between qualitative data sources, collection, analysis and interpretation? |
| (23) | Lwembe 2017 | Yes | Yes | Yes | Yes | Yes | Yes | Yes |
| (22) | Parkes  2007 | Yes | Yes | Yes | Yes | Yes | Yes | Yes |
| **RCTs (ref.)** |  | Are there clear research questions? | Does the collected data address the research questions? | Randomisation appropriately performed? | Are the groups comparable at baseline? | Are there complete outcome data? | Are outcome assessors blinded to the intervention provided? | Did the participants adhere to the assigned intervention? |
| (26) | Palmer 2021 | Yes | Yes | Yes | Yes | Yes | Yes | Yes |
| **Quantitative descriptive (ref.)** |  | Are there clear research questions? | Does the collected data address the research questions? | Is the sampling strategy relevant to address the research question? | Is the sample representative of the target population? | Are the measures appropriate? | Is the risk of nonresponse bias low? | Is the statistical analysis appropriate to answer the research question? |
| (20) | Wang 2019 | Yes | Yes | Yes | Yes | Yes | No | Can’t tell |
| (27) | Springham 2015 | Yes | Yes | Yes | No | Yes | Yes | Can’t tell |
| (19) | O’keeffe 2015 (Jigsaw) | Yes | Yes | Yes | Yes | Yes | Yes | Yes |
| **Mixed methods (ref.)** |  | Are there clear research questions? | Does the collected data address the research questions? | Is there an adequate rationale for using a mixed methods design to address the research question? | Are the different components of the study effectively integrated to answer the research question? | Are the outputs of adequately interpreted? | Are divergences between quantitative and qualitative results adequately addressed? | Do the different components of the study adhere to the quality criteria of each tradition of the methods involved? |
| (24) | Pocobello 2020 | Yes | Yes | Yes | Yes | Yes | Yes | No |
| (21) | Usman 2022 | Yes | Yes | Yes | Yes | Yes | Yes | No |
| (25) | Livingston 2013 | Yes | Yes | Yes | Yes | Yes | Yes | No |
