# Supplementary material for: Service user involvement in mental health service commissioning, development and delivery: A systematic review of service level outcomes
Source: Health Expect. 2023 Jun 8;26(4):1453–66. doi: 10.1111/hex.13788 (PMC10349231; doi:10.1111/hex.13788)
Supplement: Supplementary file 5 — Supporting information. [file HEX-26--s005.docx]

***Supporting Information 5:*** *Data extraction form*

| Study characteristics | |
| --- | --- |
| Author / year |  |
| Setting |  |
| Study design |  |
| Study aim |  |
| Number of service users |  |
| Type of service |  |
| Population served |  |
| Results | |
| Level of involvement |  |
| Method of involvement |  |
| Activities |  |
| Outputs |  |
