# Supplementary material for: Service user involvement in mental health service commissioning, development and delivery: A systematic review of service level outcomes
Source: Health Expect. 2023 Jun 8;26(4):1453–66. doi: 10.1111/hex.13788 (PMC10349231; doi:10.1111/hex.13788)
Supplement: Supplementary file 6 — Supporting information. [file HEX-26--s002.docx]

***Supporting Information 6:*** *Papers excluded at full text screening*

| **Paper** | **Reason** |
| --- | --- |
| Morant N, Lloyd-Evans B, Lamb D, Fullarton K, Brown E, Paterson B, et al. Crisis resolution and home treatment: stakeholders’ views on critical ingredients and implementation in England. BMC Psychiatry. 2017;17(1):254. | No service user involvement. |
| Cheverton J, Janamian T. The Partners in Recovery program: mental health commissioning using value co-creation. Medical Journal of Australia. 2016;204(S7):S38-S40. | Not a mental health service. |
| Robert G, Hardacre J, Locock L, Bate P, Glasby J. Redesigning mental health services: lessons on user involvement from the Mental Health Collaborative. Health Expect. 2003;6(1):60-71. | No reported service level outputs. |
| Turner A, Realpe AX, Wallace LM, Kosmala-Anderson J. A co-produced self-management programme improves psychosocial outcomes for people living with depression. Mental Health Review Journal. 2015;20(4):242-55. | Not a mental health service. |
| Lamb J, Dowrick C, Burroughs H, Beatty S, Edwards S, Bristow K, et al. Community Engagement in a complex intervention to improve access to primary mental health care for hard-to-reach groups. Health expectations: an international journal of public participation in health care and health policy. 2015;18(6):2865-79. | Not a mental health service. |
| Morrow R, McGlennon D, McDonnell C. A Novel Mental Health Crisis Service - Outcomes of Inpatient Data. The Ulster medical journal. 2016;85(1):13-7. | No service user involvement. |
| McAllister S, Simpson A, Tsianakas V, Canham N, De Meo V, Stone C, et al. Developing a theory-informed complex intervention to improve nurse-patient therapeutic engagement employing Experience-based Co-design and the Behaviour Change Wheel: an acute mental health ward case study. BMJ open. 2021;11(5):e047114. | No reported service level outputs. |
| Alliston C, Kluge C, Fudge E. Commentary on consumer and carer participation in a national mental health promotion initiative. AeJAMH (Australian e-Journal for the Advancement of Mental Health). 2009;8(3):305-14. | No reported service level outputs. |
| Abdel-Baki A, Aubin D, Morisseau-Guillot R, Lal S, Dupont M-E, Bauco P, et al. Improving mental health services for homeless youth in downtown Montreal, Canada: Partnership between a local network and ACCESS Esprits ouverts (Open Minds), a national services transformation research initiative. Early Intervention in Psychiatry. 2019;13(Suppl 1):20-8. | No reported service level outputs. |
| Kirmayer LJ, Groleau D, Guzder J, Blake C, Jarvis E. Cultural consultation: a model of mental health service for multicultural societies. Canadian journal of psychiatry Revue canadienne de psychiatrie. 2003;48(3):145-53. | No service user involvement. |
| Larkin M, Boden ZVR, Newton E. On the brink of genuinely collaborative care: Experience-based co-design in mental health. Qualitative Health Research. 2015;25(11):1463-76. | No reported service level outputs. |
| Bailey D. Letter from Birmingham. What is the way forward for a user-led approach to the delivery of mental health services in primary care? Journal of Mental Health. 1997;6(1):101-5. | No reported service level outputs. |
| Bryant W, Vacher G, Beresford P, McKay E. The modernisation of mental health day services: participatory action research exploring social networking. Mental Health Review Journal. 2010;15(3):11-21. | No reported service level outputs. |
| Dunn V. Young people, mental health practitioners and researchers co-produce a Transition Preparation Programme to improve outcomes and experience for young people leaving Child and Adolescent Mental Health Services (CAMHS). BMC Health Services Research. 2017;17:293-. | No reported service level outputs. |
| Cooper K, Gillmore C, Hogg L. Experience-based co-design in an adult psychological therapies service. Journal of Mental Health. 2016;25(1):36-40. | No reported service level outputs. |
| Wilson, J, Clarke, T, Lower, R, et al. Creating an innovative youth mental health service in the United Kingdom: The Norfolk Youth Service. Early Intervention in Psychiatry. 2018; 12: 740– 746. https://doi.org/10.1111/eip.12452 | No reported service level outputs. |
