# Supplementary material for: Service user involvement in mental health service commissioning, development and delivery: A systematic review of service level outcomes
Source: Health Expect. 2023 Jun 8;26(4):1453–66. doi: 10.1111/hex.13788 (PMC10349231; doi:10.1111/hex.13788)
Supplement: Supplementary file 7 — Supporting information. [file HEX-26--s006.pdf]

Coercion

Educating

Service users offered training so they can effectively engage in design meetings and understand methodology.

Informing

Consultation

Service users asked about their views on pre-set topics via one-off interviews.

An audit was done of service users views via postal questionnaires.

Engagement

A patient advisory committee, made up of service users and professionals met monthly to discuss issues and provide feedback on decisions made.

Service users involv[illegible] throughout service development phas[illegible] users consulted on key issues in service design e.g. types of support offered and their views were incorporated.

Co-design

Service users identified priorities for change. Under guidance of professionals, they carried out design work in small groups formed around those priorities.

Service users help to formulate action plan and review implementation plan (not involved in implementation).

Service users are part of service design meetings and have influence over key decisions. However, they were not involved in 'seeing it through'.

Co-production

Planning meetings held which aimed to share power between service users and staff. Service users are involved in and oversee service delivery.

Weekly assembly meeting where users and professionals discuss and decide on key issues regarding management of the service. Always service user majority.

Service users and staff work together from design to delivery. Service users recruited as community mental health champions. Involved in strategic decision making.
